# Mobile Phones in Research and Treatment: Ethical Guidelines and Future Directions

**DOI:** 10.2196/mhealth.4538

**Published:** 2015-10-16

**Authors:** Adrian Carter, Jacki Liddle, Wayne Hall, Helen Chenery

**Affiliations:** ^1^ School of Psychological Sciences Monash University Melbourne Australia; ^2^ UQ Centre for Clinical Research The University of Queensland Brisbane Australia; ^3^ Centre for Youth Substance Abuse Research The University of Queensland Brisbane Australia; ^4^ National Addiction Centre University of London London United Kingdom; ^5^ Faculty of Health Sciences and Medicine Bond University Robina Australia

**Keywords:** ethics, informed consent, mHealth, mobile phones, Parkinson’s disease, privacy, regulation

## Abstract

Mobile phones and other remote monitoring devices, collectively referred to as "mHealth," promise to transform the treatment of a range of conditions, including movement disorders, such as Parkinson’s disease. In this viewpoint paper, we use Parkinson’s disease as an example, although most considerations discussed below are valid for a wide variety of conditions. The ability to easily collect vast arrays of personal data over long periods will give clinicians and researchers unique insights into disease treatment and progression. These capabilities also pose new ethical challenges that health care professionals will need to manage if this promise is to be realized with minimal risk of harm. These challenges include privacy protection when anonymity is not always possible, minimization of third-party uses of mHealth data, informing patients of complex risks when obtaining consent, managing data in ways that maximize benefit while minimizing the potential for disclosure to third parties, careful communication of clinically relevant information gleaned via mHealth technologies, and rigorous evaluation and regulation of mHealth products before widespread use. Given the complex array of symptoms and differences in comfort and literacy with technology, it is likely that these solutions will need to be individualized. It is therefore critical that developers of mHealth apps engage with patients throughout the development process to ensure that the technology meets their needs. These challenges will be best met through early and ongoing engagement with patients and other relevant stakeholders.

## Introduction

Mobile phones are an increasingly common form of information and communication technology that combine mobile computing capabilities with telecommunications [1]. In 2011, there were over 6 billion cell and mobile phone subscriptions reaching 87% of the world’s population [2]; 1 in 3 subscriptions were for a mobile phone [3]. The ability to run third-party software apps on mobile phones has prompted their use in health settings to improve diagnosis and personalize health care [1]. This use of mobile phone technologies for this purpose has been termed “mHealth.”

Mobile phone apps may support an individual’s self-report of symptoms or passively record time, location, and other information using a large array of on-board instruments, such as a global positioning system (GPS), wireless local area network (WLAN; or Wi-Fi), cellular network antennae, Bluetooth, accelerometers, gyroscopes, pressure sensors, proximity-sensing magnetometers, barometers, humidity sensors, temperature sensors, and ambient light sensors [4]. Microphones and cameras may also record images and sounds in the vicinity of the phone, including personal conversations [5]. Additional external sensors may allow recording of physiological information, such as heart rate, blood pressure, glucose levels, and even brain activity using a portable brain scanner (eg, electroencephalogram) [6]. This information can be linked to other commercially available electronic databases, including Web-based platforms (eg, Facebook, Google) or government-controlled personal medical records. Algorithms may be used to organize and decode the recorded information to provide data on disease state, response to treatment, physical activity levels, falls, and tremor. Mobile phones can then send this information to research or clinical teams, which enables timely responses that were not possible using older technologies [4].

mHealth technologies are being increasingly used to help patients manage chronic, degenerative neurological diseases, such as Parkinson’s disease (PD), that produce changes in mobility, communication, mood, and independence. In the following, we use PD as an example, although most considerations discussed below are valid for a wide variety of conditions. Mobile phones and other remote sensing devices have been used with people with PD to monitor their movement at home [7-9], hand tremor [10], timing of medication and meals [7], community mobility [11], and voice patterns [12].

The ability to remotely monitor a wide range of markers of social well-being in PD is increasingly important. Medications and other treatments, such as deep brain stimulation, can improve the motor symptoms of people with PD, but these medications and treatments may not be accompanied by similar improvements in the nonmotor symptoms and quality of life outcomes [11]. Mobile phones have the potential to measure both motor and nonmotor symptoms in PD, and they can analyze large amounts of data before providing summary reports to the patient and his or her physician to guide treatment decisions [13]. The capacity of mobile phones to collect a wide range and quantity of personal information from patients raises novel and complex ethical and practical challenges that research teams and clinicians need to understand if we are to maximize the promise of the technology while minimizing any unintended harms. Analogous concerns have been examined in the context of Internet research and eHealth [14-17]. Given the additional capacity of mobile phones to collect personal information and the explosion in mHealth apps, an examination of the ethical challenges raised by the use of mobile phones in the research and treatment of persons with major neurodegenerative disorders, such as PD, is urgently needed. 

## Ethical Issues Raised by the Use of Mobile Phones for Research and in the Clinic

### Privacy, Security, and Data Ownership

Privacy is the ability to control the recording and sharing of personal information with others. This requires knowledge of what will be recorded, how it will be used and for how long, who will have access to this information, and what the risks are of discovery and misuse by third parties.

#### What Can Mobile Phones Reveal About a Person?

Geolocation technologies on a mobile phone (eg, GPS, WLAN) can reveal a range of personal information. This might include where you live, where your children go to school, whether you visit a therapist and if so how often, how often you visit drinking or gambling establishments, whether you arrive early or late to work, whether you have participated in a protest or are associated with outlawed or terrorist organizations, and other habits or routines [5,18-20]. It is possible to identify a specific individual with reasonable certainty from this information. Consequently, it may be impossible to deidentify an individual’s mobile phone data, the standard way of protecting personal privacy in research. This may be of particular relevance in PD where up to 1 in 6 patients will develop severe compulsive disorders (eg, pathological gambling, hypersexuality, compulsive shopping) as a result of their dopamine replacement therapy [21]. These behaviors can cause substantial harm to others and may come to the attention of relevant government authorities, which could then lead to criminal or civil suits [22]. The difficulty in anonymizing mobile phone data is particularly salient in a global research environment that increasingly requires the sharing of data in publicly available repositories.

The greatest threat to privacy is third-party use of data recorded, collected, and transmitted by a mobile phone. Data may come into the hands of a third party via hacking of information sent over the Internet or via Bluetooth (commonly referred to as “sniffing”); legal interception by government agencies (eg, subpoena); incidental discovery by someone accessing the phone; or by telecommunication companies and cloud storage providers, for example, Internet service provider (ISP), Google, Amazon, who may claim ownership of the data recorded by or transmitted through their networks [23].

##### Subpoena and Government Interception

Researchers and clinicians can only provide limited guarantees on privacy protection. For instance, data collected on mobile phones may be subpoenaed as part of legal proceedings in civil (eg, divorce, litigation) or criminal cases. This includes both the data collected on the phone itself or data and their analysis held by researchers or clinicians. Researchers and clinicians are obliged to hand over such private information when subpoenaed.

Mobile phones are more prominent in the public domain than in traditional office-based pen-and-paper or desktop computer research kept within the offices of researchers or clinicians. The information collected via mobile phones is more liable to be encountered by third parties who are not involved in the research or clinical care. The legal subpoena of clinical data is possible within any research setting. However, a person’s participation in a research study is more likely to become known to authorities through the presence of an app on their mobile phone [20]. The simple discovery of an mHealth app on a phone may be enough to disclose personal information that users may wish to keep private, such as their having a neurodegenerative disorder or medication-induced addiction.

##### Hacking and Third-Party Data Ownership

The security of data collected via mobile phones cannot be guaranteed. Hacking of personal data from mHealth apps has resulted in medical identity theft and significant financial losses [23], and such data have been used in the courts [24]. mHealth apps are not required to adhere to strict privacy regulations, such as the US Health Insurance Portability and Accountability Act Privacy Rule, and therefore may be stored and transferred using methods that are less secure than those normally required of electronic medical data [23]. The data transmitted may include usernames, passwords, and other personal information that may enable forms of identity theft.

Encryption is essential for the storage and transmission of data using mobile phones; however, a recent study found that many mHealth apps do not use encryption when transferring data [23]. Even with modern encryption methods, data may be accessible to hackers and/or government authorities. The recent incidences of high-profile mobile phone hacking in the United States and United Kingdom illustrate the vulnerability of mobile phone users to privacy violations [25]. There are also potential privacy violations from computer malware and virus programs that exploit vulnerabilities in how data are stored on the device or from malevolent app developers who steal data for commercial or criminal interests [23,26]. Developers can take steps to ensure that data collected by an mHealth app are not available (eg, via data logs, SD card storage, exported, and side channels) to other apps or programs contained on the phone [23]. As He et al [23] demonstrate, most mHealth apps currently available do not take the necessary security measures to protect an individual’s privacy.

It is also not clear who owns the data in research and clinical settings. Mobile phone data may be transmitted to the research team or clinician via ISP or telecommunication companies. These companies often record metadata as well as the data transferred over their networks, and may sell them to other third parties. Government agencies may also obtain access to this information, as revealed by Edward Snowden in the recent National Security Agency affair [27].

### Obtaining Informed Consent

Before using mHealth technologies, clinicians and researchers need to seek and obtain the informed consent from participants. Participants need to be informed of the risks and benefits of using mHealth technologies and must have the capacity to understand these risks and make a free and uncoerced decision about whether to participate [28]. A challenge in mHealth is communicating the complex nature of the risks raised by this technology and negotiating the risks that individuals are willing to face. How much input should people have over what they wish to have recorded and shared? How will the data be used, where will it be stored and in what form, how will it be shared, and for how long? Participants must also be made aware of what will happen to their data once their study participation is complete.

Researchers and clinicians using mobile phone technologies need to develop consent processes that actively engage individuals in their own privacy decision making as much as possible. Understanding the risks posed by third-party access to their personal health data can be difficult to communicate given the complexity of mHealth technologies. Meeting these ethical challenges will require technology developers to create apps that assist participants in understanding their participation and give them as much say as possible in what is shared and with whom. This may involve trade-offs between, on the one hand, maximizing the data obtained and what may be done with it, and, on the other hand, enabling participants to control what they consent to and how their data may be used and stored.

As part of the consent process, researchers and clinicians need to inform participants about the circumstances in which they are obliged to disclose the participants’ personal information. This potentially includes information that can pose an immediate and likely threat to themselves or to others (eg, suicide, homicide). Patients must also be informed of the potential for identification of “incidental findings,” specifically clinically significant information related to their health, such as cognitive impairment and dementia, depression and other psychiatric disorders, or medication abuse or other compulsive behaviors that may benefit from additional treatment. Patients should be informed about the processes in place to deal with potential incidental findings and provide them with further information and access to clinical treatment should any emerge. Participants should also be given the opportunity to indicate whether they would like to be told about the presence or absence of incidental findings.

The potential for mobile phones to record conversations about third parties or bystanders raises additional ethical and legal concerns. For example, mobile phones may record conversations to examine the impact of PD on patients’ speech and socialization [11,12]. Researchers may obtain consent from the research participant to have their conversations recorded but they cannot easily obtain consent from bystanders (eg, friends and family) who may also be recorded via the mobile phone. The recording and/or communicating of third-party conversations (even to a secure portal) is illegal in some jurisdictions. Researchers may therefore need to develop methods for ensuring that bystanders are able to consent to having their conversations recorded. One option would be to provide an alert on the mobile phone indicating to the participant that a recent conversation has been audio recorded so that they can ask third parties to consent to the recording. An opt-in approach could be applied where the conversation is only sent to researchers or clinicians if the third-party individuals agree. This would further burden the participant who would have to disclose their mHealth participation and, therefore, possibly their clinical condition to obtain the third party’s consent. The opt-in approach also obtains consent after the fact, and therefore, may be inadequate in some jurisdictions.

### Storing and Sharing Mobile Phone Data

How mobile phone data are stored and shared can have important implications for privacy risk. Developers may choose to create a secure “vault” on the device that is physically transferred to researchers or clinicians at predetermined intervals. This allows greater control of data and protection for participants, but at the cost of reduced flexibility and timeliness. It could limit the benefits that a patient or participant may derive from the ability to self-manage their condition.

The most clinically and scientifically useful approach is to periodically upload the data electronically (eg, via 3G or wireless networks) to researchers or clinicians. This allows for instantaneous transfer of data, but provides less privacy protections as it involves transmitting data over third-party networks. Developers will need to decide where and when data are uploaded (eg, only at certain locations or times, such as from a home Wi-Fi network) as opposed to routinely uploading via public Wi-Fi or mobile networks. Developers will need to reach a balance between maximizing clinical or scientific utility while minimizing the risk to privacy. Solutions will vary depending on how the technology is being employed.

Developers will also need to consider what data to collect and transmit from the mobile phone. The ability to collect as much raw data as possible maximizes the information that a research and clinical team can extract. It also increases the potential risk to the participant. A balance needs to be struck between maximizing scientific and clinical utility and protecting privacy. It is preferable to only collect data sufficient for the purpose of the study or clinical intervention (eg, postcode or distance from home), rather than collecting all data routinely. By collecting only the minimum amount of information, app developers can help participants maintain control over their raw data [20].

Developers also need to make data comprehensible to the participant before they are sent or before consent is given to share the data. Developers should provide easily understandable visual information to participants about where their information will go, who will have access to it, and for how long. This will greatly increase participants’ understanding of the risks involved with this technology and enable them to make more informed decisions. Such processes will help participants to see what their sharing policies are and what the results of these policies will be [20].

### Communication of Clinical or Research Results

A critical decision in the clinical or research use of mobile phones is when and what to tell research participants or patients about their data. The communication of clinically meaningful feedback to participants about their data maximizes the benefits for participants and mitigates the harm from potential privacy violations. To be clinically meaningful, the findings must be scientifically robust. The ethical obligation to share an individual’s data with them is more acute in the clinical setting where the explicit aim is to facilitate better clinical management of the disease, and there is strong evidence to support the clinical claims being made. Feedback is also provided by a qualified individual responsible for the patient’s clinical management. In research, the clinical relevance of data is, by definition, less certain because the research aim is to establish an evidence base for clinical intervention. In research, providing individualized clinical feedback from the data is inappropriate, and may lead to additional harm if the clinical advice is misleading or provided by nonprofessionals.

The provision of immediate and useful information by appropriately qualified clinicians can potentially better enable individuals to manage their health and improve the clinician/patient relationship by empowering patients to take more control over their health and well-being [29,30]. However, to serve this purpose the information collected by the mobile phone needs to be presented in ways that are meaningful, accurate, and easily understood. Well-designed platforms that provide such feedback to patients will maximize the potential for individuals to self-manage their health and well-being. Participants may also be assisted to access effective clinical services for the treatment of any symptoms identified in the data collected via their mobile phone. Developers and researchers may also wish to identify opportunities in the app in which participants can receive information that will enable them to make healthier choices. Research is needed to establish what impact the provision of clinical information recorded on mobile phones may have on a person’s behavior. Communication of deteriorating motor symptoms, for example, could adversely impact on well-being and health-related behaviors. See Eonta et al [31] for practical guidance on the ethical communication of clinical information to patients using mobile phones and other mHealth technologies.

### Access to mHealth Technology

While there has been a rapid growth in mobile phone coverage in recent years, some segments of the population lack access. People from lower socioeconomic groups may not be able to afford a phone capable of supporting the app or connecting with mobile or Internet networks required to transmit potentially large volumes of data. There is an ethical imperative not to exclude these patients from benefiting from mHealth monitoring.

Disease-related impairments may also interfere with the effective use of these technologies. PD may impair both fine motor and speech skills, therefore creating difficulty in accessing touch screens or voice-based interaction with mobile phones. Designers of mHealth apps must consider ways of reducing the effects of these cognitive, motor, or other impairments. Having a choice on how to interact with an app is one approach (eg, voice-activated, image- or text-based interface). People with PD who experience fine motor difficulties may be assisted by reducing the sensitivity of the screen to repeated touch, appropriate spacing and sizing of buttons, use of swipe on-screen keyboard, and external devices such as a stylus. People with voice-related difficulties may be assisted by a plug-in microphone and software that can be trained to an individual’s voice (eg, Dragon Dictate).

Given the complex array of symptoms and differences in comfort and literacy with technology, it is likely that these solutions will need to be individualized. It is therefore critical that developers of mHealth apps engage with patients throughout the development process to ensure that the technology meets their needs. This will assist developers, clinicians, and research teams to target the symptoms that are of greatest concern to patients. Developers should use reference groups (eg, consumer, family, industry, health care professional) to anticipate challenges in developing technology that meets these challenges [11]. As one patient reported in regard to the use of mobile phones for diabetes, “It’s not just about blood glucose results and HbA1c results, it’s about how people feel and, perhaps, how their mood may affect their glucose levels” [29]. A summary of the recommendations for managing these ethical challenges is presented in Table 1.

**Table 1:** Ethical recommendations for the use of mobile phones in the clinic and laboratory.

Ethical issue	Recommendation
Anonymity and de-identification	In many cases, deidentification is not possible. Developers must communicate this to users when obtaining consent.
Third-party use of data	Individuals should be informed about the risk of third parties accessing data collected on mobile phones, either via hacking, legal interception (eg, subpoena), incidental discovery by someone accessing the phone, or by telecommunication companies (eg, ISP provider, Google) that may claim ownership of the data recorded by or transmitted through their networks.
Storage and transmission of data	The risk of privacy violations can be minimized by thoughtful consideration of how the data are stored locally on the phone or transmitted to the research or clinical team. Apps can be placed in secure vaults on mobile phones to minimize incidental discovery. Developers should also record and transmit the minimal amount of data necessary for the purpose of the app.
Obtaining informed consent	Participants need to be informed about the risks and benefits of using mHealth technologies and must have the capacity to understand these risks and make a free and uncoerced decision about whether to participate. Researchers and clinicians should employ visual aids that communicate the complex nature of the risks posed by mHealth. Developers should maximize opportunities for users to control what data are shared with the clinical/research team, such as through the use of pop-up messages asking whether they consent to specific information being shared.
Communication of clinically relevant results	Feedback of clinically relevant information should be provided by a qualified health care professional and when there is strong empirical evidence to support the findings. Users should be informed at the point of consent what information may be uncovered by mobile phones and whether they will be informed about these findings. Patients should also be assisted with accessing necessary clinical services as a result of the findings.
Access to mHealth technologies	Efforts should be undertaken to prevent individuals benefiting from advances in mHealth as a result of their socioeconomic status or physical or mental impairments.
Active engagement with patients	Developers should use reference groups (eg, consumer, family, industry, health care professional) throughout the development process to ensure that the technology meets participants’ needs.
Regulation of mHealth products	mHealth technologies should be rigorously evaluated to demonstrate their safety and effectiveness before the widespread rolling out of mHealth apps and associated products.

### Regulating and Evaluating mHealth Apps and Products

An explosion in mHealth research [32] has led to a rapid proliferation of small pilot or seed programs, many of which lack scientific evidence of efficacy on which to base clinical use. There is limited evidence on the effectiveness, or safety, of mHealth apps as a self-management tool for improving health [29]. Hence, there is an urgent need to evaluate their effectiveness via randomized controlled trials before the widespread rollout of mHealth apps and associated products (eg, brain or heart monitors) [33]. This need was recognized by the World Health Organization and other leading agencies responsible for implementing medical products in the Bellagio call to action on global eHealth evaluation that called for rigorous evaluation “to generate evidence and promote the appropriate integration and use of technologies...to improve health and reduce health inequalities” [34].

It is imperative that the use of mHealth apps by researchers, clinicians, universities, and hospitals is based on rigorous evaluations of their effectiveness and safety [29], using the CONSORT-EHEALTH checklist [35] and GRADE framework [32]. Policy makers should not be seduced by the hype and promise of mHealth technology. The prima facie simplicity and cost effectiveness of mHealth solutions may blind decision makers to the lack of robust empirical evidence that is needed to justify their routine use [29]. Premature implementation of untested mHealth interventions may result in failed projects, wasted resources, and poorer health outcomes for patients.

There is currently no regulation of mHealth devices or apps and no guarantee that they provide clinically accurate information. The Food and Drug Administration (FDA) recently released guidelines for how they intend to regulate the marketing of mHealth apps that meet the definition of medical devices (ie, those “whose functionality could pose a risk to a patient’s safety if the mobile app were to not function as intended”) [36], although these recommendations are currently nonbinding and do not prevent apps from being made available. The FDA has recently approved the marketing of an mHealth app to continuously monitor glucose levels [36].

However, most apps are made available to patients directly via publicly available app stores, without passing through regulatory gatekeepers to ensure their safety and effectiveness. The clinical use of these devices and apps needs to be regulated in the same way as any other medical or psychotherapeutic intervention. This is particularly important given the influence of vested commercial interests that may push for quick rollout and be more concerned with growing markets than improving global health [37]. The regulation of mHealth products would also help to minimize privacy violations through malware or other computer-based viruses. The development of a Web-based approval system for verifying the quality of mHealth apps, such as the Health on the Net Foundation Code of Conduct, or National Health Service app stores, such as the United Kingdom Health Apps Library, may be useful for ensuring the quality of mHealth apps.

Regulators will also need to deal with the increasingly globalized nature of mHealth research. Traditional processes of institutional ethics approval and recruitment provide significant barriers to conducting mHealth research that crosses national borders and involves numerous agencies. Ethical oversight of mobile phone research will need to evolve in order to allow it to happen, and to prevent it from being done with insufficient ethical oversight by commercial entities (eg, Apple, Facebook, Google). Failure to do so may leave these companies with a vastly superior understanding of health and behavior than researchers, governments, and policy makers. Unlike publicly funded research, these findings will be protected by commercial-in-confidence and trade secrets laws.

## Conclusions

Mobile phones and other remote monitoring devices have the potential to provide researchers with access to unprecedented volumes of clinically relevant data on patients’ quality of life and psychosocial functioning, movement at home, in the community, and social integration. Neurologists and other treating clinicians will get real-time measures of disease progression and the impact of medication over periods not previously possible. The promise of this technology—the ability to collect, analyze, and communicate vast amounts of personal data almost immediately to research and clinical teams—also poses new and unique ethical and technical challenges that need to be managed if we are to realize the promise while minimizing potential risks of harm. While the ethical issues of privacy, consent and equity are not unique to mHealth, specific solutions are needed that address the particular ethical challenges raised my mobile phone technologies. The development of mobile phone apps that optimally address these challenges will require early and ongoing engagement with patients and other relevant stakeholders.

## Abbreviations

APCN: Asia-Pacific Centre for Neuromodulation
FDA: Food and Drug Administration
GPS: global positioning system
ISP: Internet service provider
PD: Parkinson’s disease
WLAN: wireless local area network
